# Symbiosis Studies in Microbial Ecology

**DOI:** 10.1264/jsme2.ME3103rh

**Published:** 2016-09

**Authors:** Yoichi Kamagata, Takashi Narihiro

**Affiliations:** 1 Bioproduction Research Institute, National Institute of Advanced Industrial Science and Technology (AIST)Tsukuba, Central 6, Tsukuba, Ibaraki 305–8566Japan

Symbiosis has long fascinated scientists: how are microbe and microbe interacting together, and how are microbes associated with host insects, animals and plants? Those studies provide deep insights into co-evolution of life showing how microorganisms have created specific niches closely associated with different organisms, how they have sustained the life of host organisms and how microbes as endosymbiont or even host organisms have adapted their way of living in the course of symbiotic interaction. Recent issues of Microbes and Environments provide a variety of intriguing symbiosis studies. Specifically, it has published very interesting papers on symbionts of stinkbugs, aphids, cockroaches, honey bees, crab, termites, ciliates, and so forth.

A plethora of phytophagous stinkbugs harbor symbiotic bacteria in a specific midgut region composed of numerous crypts. Among the five superfamilies of the infraorder Pentatomomorpha, most members of the Coreoidea and Lygaeoidea are associated with a specific bacterial group of the genus *Burkholderia*, often referred to as the “stinkbug-associated beneficial and environmental (SBE)” group (10–12), which is not vertically transmitted, but acquired from the environment every host generation. In addition to these two stinkbug groups, the family Largidae of the superfamily Pyrrhocoroidea also possesses a *Burkholderia* symbiont. Takeshita *et al.* (29) demonstrated that the largid species are consistently associated with the “plant-associated beneficial and environmental (PBE)” group of *Burkholderia*, which are phylogenetically distinct from the SBE group, and that they keep symbiosis through the environmental acquisition of the bacteria. Since the superfamilies Coreoidea, Lygaeoidea, and Pyrrhocoroidea are monophyletic in the infraorder Pentatomomorpha, it is plausible that the symbiotic association with *Burkholderia* evolved at the common ancestor of the three superfamilies. But the results strongly suggest that a dynamic transition from the PBE to SBE group, or vice versa, occurred in the course of stinkbug evolution.

Kuechler *et al.* (15) investigated four species of the stenocephalid genus *Dicranocephalus*, another coreoid family, fluorescence *in situ* hybridization (FISH) and transmission electron microscopy (TEM) revealed the typical arrangement and ultrastructures of midgut crypts and gut symbionts. Cloning and molecular phylogenetic analyses of bacterial genes showed that the midgut crypts of all species are colonized by *Burkholderia* strains, which were further assigned to different subgroups of the genus *Burkholderia*. In addition to the SBE-group *Burkholderia*, a number of stenocephalid symbionts were found to be affiliated with a novel clade containing *B. sordidicola* and *B. udeis*, suggesting a specific symbiont clade for the *Stenocephalidae*. The study indicates ongoing evolution of symbiont associations in the stinkbug-*Burkholderia* interaction.

The narrative of stinkbugs’ endosymbionts continues. The stinkbug *Cavelerius saccharivorus*, which harbors *Burkholderia* species capable of degrading the organophosphorus insecticide, fenitrothion, were identified on a Japanese island in farmers’ sugarcane fields that have been exposed to fenitrothion (26, 27). In the study by Tago *et al.* (28), they analyzed the composition and abundance of degraders in sugarcane fields on the island. Distribution of fenitrothion degraders mostly belonging to the genus *Burkholderia* varied due to differences in insecticide treatment histories. The strains related to the stinkbug symbiotic group predominated among the degraders, indicating a selection for this group in response to fenitrothion. Degraders were residing in sugarcane stems, leaves, and rhizosphere in fields that were continuously exposed to fenitrothion. They concluded that plants and the rhizosphere constituted environmental reservoirs for stinkbug symbiotic degraders.

Aphid is a well-known and long studied insect that harbors *Buchnera aphidicola* as the primary endosymbiont with which it maintains an obligate mutualistic symbiotic relationship (14). Insects also maintain facultative symbiotic relationships with secondary symbionts, and *Serratia symbiotica* is the most common in aphids. Martínez-Díaz *et al.* (18) examined two closely related aphids, *Cinara tujafilina* and *C. cedri* and found that both *B. aphidicola* strains have similar genome sizes and gene contents, the genomes of the two *S. symbiotica* strains were remarkably different. The *Serratia* strain in *C. cedri* (SCc) has the smallest genome known for this species, while that in *C. tujafilina* (SCt) possesses a larger genome. Over two years of investigation using two geographically close populations, they found a positive correlation between both endosymbiont densities and average daily temperatures in the *C. tujafilina* population. *S. symbiotica* SCt may retain some protective role against heat stress.

Cockroaches are parasitized by thelastomatid nematodes, which live in an obligate manner in their hindgut and interact with the resident microbial community (19). Vicente *et al.* (31) analyzed the gut microbiome of *Periplaneta fuliginosa* and *P. americana* to investigate natural and artificial infection by thelastomatid nematodes. They examined the complete gut microbiome (fore-, mid-, and hindgut) of lab-reared *P. fuliginosa* naturally infected with the parasitic nematode *Leidynema appendiculatum* and those that were nematode-free. Results revealed that the fore- and midgut of naturally infected and nematode-free *P. fuliginosa* have close microbial communities, which is in contrast with hindgut communities; the hindgut communities of both cockroaches exhibit higher microbial diversities in the presence of their natural parasites and marked differences were observed in the abundance of the most representative taxa, *Firmicutes*, *Proteobacteria*, and *Bacteroidetes* showing that multitrophic interactions in the cockroach gut affected by the thelastomatid nematodes.

Honey bees (*Apis mellifera*) are crop pollinators and are important for effective food production. The honey bee gut microbiota is mainly host specific, with only a few species being shared with other insects (16). It remains unclear how environmental/dietary conditions affect the microbiota within a honey bee population over time. Ludvigsen *et al.* (17) characterized the composition of the midgut/pyloric microbiota of a honey bee apiary throughout a season. Monthly sampling of a demographic homogenous population of bees was performed between May and October, with recording of the honey bee diet. They showed that a marked increase in α-diversity was detected between May and June. They found that four distinct phylotypes belonging to the *Proteobacteria* dominated the microbiota, and these displayed major shifts throughout the season. *Gilliamella apicola* dominated the composition early on, and *Snodgrassella alvi* began to dominate when the other bacteria declined to an absolute low in October. In vitro co-culturing revealed that *G. apicola* suppressed *S. alvi*. No shift was detected in the composition of the microbiota under stable environment/dietary conditions between November and February. These results indicate that environmental/dietary changes may trigger the shifts in the honey bee midgut/pyloric microbiota throughout a season.

Researchers are also taking a closer look at symbionts in the deep sea floors with a different perspective. In deep-sea hydrothermal environments, most invertebrates associate with dense populations of symbiotic microorganisms in order to obtain nutrition (2). The molecular interactions between deep-sea animals and their symbionts are poorly understood. Hemagglutinins/lectins, which are carbohydrate-binding proteins, have recently been reported to play important roles in a wide array of biological processes, including the recognition and control of non-self materials. Fujiyoshi *et al.* (3) assessed hemagglutination activity in the serum of a deep-sea vent endemic crab, *Shinkaia crosnieri*, which harbors chemo-synthetic ectosymbionts on its plumose setae. Horse and rabbit erythrocytes were agglutinated using this serum. Agglutinating activity was inhibited by eight kinds of sugars and several divalent cations and remained detectable even after heating the serum at 100°C for 30 min. By using fluorescently labeled serum, they showed that deep-sea crab serum components bound to the ectosymbionts even in the presence of sugars. This report is the unique assessment of a deep-sea vent endemic crab showing the possibility of a non-lectin-mediated symbiont-host interaction.

Termites are one of the most interesting animals for their gut community structure. Gut microbiota greatly contribute to digestion of cellulosic materials to feed host. The microbiota of many phylogenetically lower termites is dominated by the cellulolytic flagellates of the genus *Trichonympha*, which are consistently associated with bacterial symbionts. In the case of *Endomicrobia*, an unusual lineage of endosymbionts of the *Elusimicrobia* phylum that is also present in other gut flagellates, previous studies (4, 7, 9) have documented strict host specificity, leading to the cospeciation of “*Candidatus* Endomicrobium trichonymphae” with their respective flagellate hosts. However, it is currently unclear whether one *Trichonympha* species is capable of harboring more than one *Endomicrobia* phylotype. Zheng *et al.* (35) selected single *Trichonympha* cells from the guts of *Zootermopsis nevadensis* and *Reticulitermes santonensis* and characterized their *Endomicrobia* populations based on internal transcribed spacer (ITS) region sequences. They found that each host cell harbored a homogeneous population of symbionts that were specific to their respective host species, but phylogenetically distinct between each host lineage, corroborating cospeciation being caused by vertical inheritance.

Anaerobic ciliates are known to harbor endosymbiotic bacteria and archaea (24, 25, 34). Hirakata *et al.* (5) investigated the prokaryotic community structure of the anaerobic ciliate, *Metopus* sp. using rRNA sequencing, FISH and TEM. *Metopus* sp. cells were taken from anaerobic granular sludge in a domestic wastewater treatment plant and anoxically cultivated for 7 d. 16S rRNA gene sequences from the prokaryotes *Methanoregula boonei* and *Clostridium aminobutyricum* were abundantly detected in *Metopus* ciliates. The FISH analysis demonstrated that these prokaryotes were localized within *Metopus* cells. They were successful in identifying *M. boonei*- and *C. aminobutyricum*-like prokaryotes as novel endosymbionts of *Metopus* ciliates. As the function of endosymbionts in anaerobic ciliates are largely unknown, the further studies will uncover the ecophysiology of those bacterial and archaeal symbionts.

In the massive sequencing era, the *de novo* assembly of an endosymbiont genome remains a challenge when host and/or mitochondrial DNA sequences are present in a dataset and hinder the assembly of the genome (21, 23, 30). By focusing on the traits of genome evolution in endosymbionts, Kinjo *et al.* (13) developed and investigated a genome-assembly strategy that consisted of two consecutive procedures: the selection of endosymbiont contigs from an output obtained from a *de novo* assembly performed using a TBLASTX search against a reference genome, named TBLASTX Contig Selection and Filtering (TCSF), and the iterative reassembling of the genome from reads mapped on the selected contigs, named Iterative Mapping and ReAssembling (IMRA), to merge the contigs. To validate this approach, they sequenced two strains of the cockroach endosymbiont *Blattabacterium cuenoti* and applied this strategy to the datasets. TCSF was determined to be highly accurate and sensitive in contig selection even when the genome of a distantly related free-living bacterium was used as a reference genome. Furthermore, the use of IMRA markedly improved sequence assemblies: the genomic sequence of an endosymbiont was almost completed from a dataset containing only 3% of the sequences of the endosymbiont’s genome. This approach together with other bioinformatics ways (6) will open the new door to scrutinize the physiological and ecological functions of symbionts in a precise manner.

Thanks to the contributions of active scientists, Microbes and Environments has been publishing a number of symbiosis papers. Not only symbionts in invertebrates and protozoa, but it has published many studies on plant-microbe interactions (1, 8, 20, 22, 32, 33, 36). Undoubtedly, these studies are obviously the pivotal topics in microbial ecology since they are ultimately addressing “why we are living together” in environments.
